# Epidemiology of Antibody-Positive Autoimmune Encephalitis in Southwest China: A Multicenter Study

**DOI:** 10.3389/fimmu.2019.02611

**Published:** 2019-11-12

**Authors:** Yixue Gu, Min Zhong, Liang He, Wei Li, Yuanyuan Huang, Jing Liu, Yangmei Chen, Zheng Xiao

**Affiliations:** ^1^Department of Neurology, First Affiliated Hospital of Chongqing Medical University, Chongqing, China; ^2^Department of Neurology, Children's Hospital of Chongqing Medical University, Chongqing, China; ^3^Department of Neurology, The Second Affiliated Hospital of Chongqing Medical University, Chongqing, China; ^4^Department of Neurology, Daping Hospital & Research Institute of Surgery, The Army Military Medical University, Chongqing, China; ^5^Department of Neurology, Southwest Hospital, The Third Military Medical University (Army Medical University), Chongqing, China; ^6^Department of Neurology, Chongqing Three Gorges Central Hospital, Chongqing, China

**Keywords:** autoimmune encephalitis, epidemiology, age, sex, neuronal autoantibodies

## Abstract

In recent years, as an increasing number of neuronal autoantibodies have been detected and used for clinical diagnosis, clinicians have become more aware of autoimmune encephalitis, causing its reported incidence to trend upward over several years. To date, however, there has been no large-scale epidemiological survey of autoimmune encephalitis in adults and children, and its epidemiological characteristics remain unclear. Six main types of antibodies are detected and used to diagnose autoimmune encephalitis in Chongqing, Southwestern China: anti-NMDA receptor antibody, anti-GABA_B_ receptor antibody, anti-LGI1 antibody, anti-CASPR2 antibody, anti-AMPA1 receptor antibody, and anti-AMPA2 receptor antibody. From January 2012 to February 2018, 189 patients at six general hospitals in Chongqing were diagnosed with autoimmune encephalitis and were positive for neuronal autoantibodies. In this report, the epidemic situation and the antibody distribution among these patients are analyzed and described in detail. The differences in disease severity among different ages and between the sexes are evaluated, and the correlation between antibody titer and disease severity is also assessed.

## Introduction

Encephalitis has high incidence and mortality rates worldwide (1), with a reported mortality rate of 8–18.45% (2–4). The term autoimmune encephalitis (AE) refers in general to a large group of diseases caused by an antigen-antibody reaction by the immune system to the central nervous system (5). The main clinical characteristics of AE are acute or subacute seizures of epilepsy, cognitive impairment, and mental symptoms. The disease spectrum of AE has been expanding since the first case of teratoma-related anti-N-methyl-D-aspartate (NMDA) receptor encephalitis was reported in 2007 (6). With the continuous progress and implementation of detection methods, a growing number of cases of AE with positivity for different autoantibodies have been diagnosed and reported. As an important cause of encephalitis, autoimmunity is receiving increasing attention from medical staff.

Investigations have shown that AE affects the quality of life of those affected and imposes a serious economic burden on both patients and society (7). Because the clinical manifestations of AE are very complex, the condition is difficult to diagnose, although early intervention is important for improving the prognosis of these patients (8). Neuronal autoantibodies are key for the diagnosis of AE, and changes in antibody titer are closely related to the clinical course (9). Neuronal autoantibodies identify subtypes of AE and help clinicians detect cases with atypical clinical manifestations. Therefore, antibody measurement is a critical step in the diagnosis of AE (5).

To date, there have been few epidemiological investigations of AE, and there are currently no data from large-scale epidemiological investigations. Thus, the epidemiological characteristics of the condition are still unclear. Six main types of antibodies are detected and used for the diagnosis of AE in Chongqing, Southwestern China: anti-NMDA receptor (NMDAR) antibody, anti-gamma-aminobutyric acid-B receptor (GABA_B_R) antibody, anti-leucine-rich glioma-inactivated 1 (LGI1) antibody, anti-contactin-associated protein-like 2 (CASPR2) antibody, anti-α-amino-3-hydroxy-5-methyl-4-isoxazolepropionic acid 1 (AMPA1) receptor antibody, and anti-α-amino-3-hydroxy-5-methyl-4-isoxazolepropionic acid 2 (AMPA2) receptor antibody. In this study, the epidemiological characteristics of 189 patients with AE and antibody positivity were analyzed according to the diagnostic criteria of AE (5). The findings will provide clinicians with an improved understanding of the epidemiological characteristics of AE and contribute to speeding the diagnostic process and improving patient prognosis.

## Methods

### Study Population

Data were collected from six large general hospitals in Chongqing, Southwestern China. From January 2012 to February 2018, 189 patients with AE were diagnosed with antibody positivity. The patients' medical records, laboratory results, cost information, and prognoses were reviewed and registered by a neurologist.

### Inclusion Criteria

According to the AE diagnostic criteria published in *The Lancet Neurology* (5), the following four criteria were used, along with positivity for neuron surface antibodies: (1) subacute onset (rapid progression over <3 months); working memory deficits, epilepsy, or psychiatric symptoms related to the limbic system; (2) bilateral brain abnormalities highly restricted to the medial temporal lobe on T2-weighted fluid-attenuation inversion recovery MRI; (3) at least one of the following: 1) an increase in the number of cerebrospinal fluid (CSF) cells (white blood cell count exceeding 5/mm^3^) 2) EEG indicating epilepsy or slow-wave activity in the medial temporal lobe; and (4) reasonable exclusion of other diseases.

### Exclusion Criteria

The exclusion criteria for the study were as follows: (1) no lumbar puncture CSF examination performed or incomplete clinical data from the period of hospitalization; (2) central nervous system infection caused by specific intracranial pathogens; (3) thyroid disease, a recent history of thyroid hormone replacement, or a lack of test results on thyroid function and antibodies; (4) an immunosuppressed state (including long-term immunosuppressive therapy due to chemotherapy, organ transplantation, or cancer); and (5) loss to follow-up.

This study was approved by the ethics committees of the six participating hospitals. All patients or their families were informed of the study and gave signed consent to allow the use of their medical records for the study.

### Antibody Detection Methods

Six hospitals sent CSF and serum to the same laboratory, which began to detect AE-related antibodies in June 2011. Six types of antibodies were detected: anti-NMDAR antibody, anti-GABA_B_R antibody, anti-LGI1 antibody, anti-CASPR2 antibody, anti-AMPA1 receptor antibody, and anti-AMPA2 receptor antibody. The laboratory used indirect immunofluorescence (IIF) assays for antibody detection. A cell-based assay (CBA) with high specificity and sensitivity was used to analyze the CSF and serum of each patient. The initial dilution titers of CSF and serum were 1:1 and 1:10, respectively. Serum antibody titers were considered weakly positive at 1:10, positive at 1:32 to 1:100, and strongly positive at 1:320. The titers of CSF antibodies were considered weakly positive at 1:1, positive at 1:3.2 to 1:10, and strongly positive at 1:32 or above.

### Statistical Analysis

The classification variables are described as percentages, and the characteristics of each subgroup are represented by the median. The chi-squared test or Fisher's exact test was used to compare differences among the subsets of classification variables. An independent-sample *t*-test was employed to compare differences among subgroups of continuous variables. The Wilcoxon signed-rank test was applied to compare differences among subgroups of hierarchical data. Spearman correlation analysis was used to analyze correlations among classified variables. SPSS 25.0 software was utilized to analyze and sort the data, with *P* < 0.05 indicating a significant difference.

## Results

### Baseline Demography and Incidence Trends

From January 2012 to 2018, 189 patients with AE and neuronal autoantibody positivity were diagnosed at six large general hospitals in Southwestern China. Samples from 457 patients with suspected AE were analyzed for antibodies, with a positivity rate of 41.36%. Five patients died, giving a mortality rate of 2.65%. In terms of prognosis, the Glasgow Outcome Scale (GOS) was used for evaluation. Those discharged with a score of >4 had a good prognosis, those discharged with a score of 2–3 had a poor prognosis, and those with a score of 1 died. The prognosis was good in 161 cases (85.19%). There were 41 cases (21.69%) in spring, 42 cases (22.22%) in summer, 59 cases (31.22%) in autumn, and 44 cases (24.87%) in winter. The median hospitalization time was 21 days, and the median hospitalization expenses were 4623.35 USD.

Among the 189 patients, females (116, 61.38%) outnumbered males (73, 38.62%) by a statistically significant margin (χ^2^ = 9.783, *P* = 0.002). The youngest patient was 1 year and 6 months old, and the oldest was 70 years old; the median age was 16 years. Among all patients, 99 (52.38%) were under 18 years old, 58 (30.69%) were 18–44 years old, 26 (7.72%) were 45–59 years old, and 6 (3.17%) were aged 60 years or older. The vast majority (83.07%) of patients were children and young adults (χ^2^ = 213.556, *P* = 0.000).

**Figure 1:**
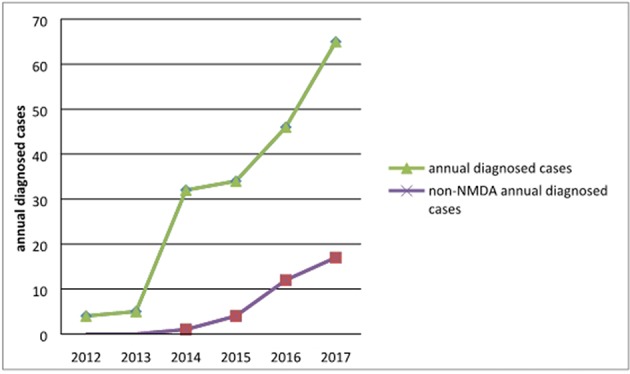
Trends in the numbers of annual diagnosed cases of AE and non-NMDAR encephalitis.

Of the 189 patients, 4 were diagnosed in 2012, accounting for 2.12% of all patients; 5 in 2013, accounting for 2.65%; and 32 in 2014, accounting for 16.93%; there were 34 cases (17.99%) in 2015 and 46 (24.34%) in 2016. In 2017, there were 65 cases, accounting for 34.39%. Three cases were confirmed in January and February 2018. The annual number of confirmed cases trended upward, as shown in Figure 1. With the development of diagnostic methods, an increasing number of autoimmune antibodies are used in clinical diagnosis. Using non-NMDAR antibodies, 1 case was diagnosed in 2014, 5 in 2015, 12 in 2016, 17 in 2017, and 2 in 2018, as shown in Figure 1.

### Antibody Distribution

Among the 189 patients, 153 (80.95%) were positive for anti-NMDAR antibody, 14 (7.41%) for anti-GABA_B_R antibody, 9 (4.76%) for anti-LGI1 antibody, 5 (2.65%) for anti-CASPR2 antibody, 3 (1.59%) for both anti-NMDAR and anti-GABA_B_R antibodies, 3 (1.59%) for both anti-LGI1 and anti-CASPR2 antibodies, 1 (0.53%) for both anti-NMDAR and anti-CASPR2 antibodies, and 1 (0.53%) for both anti-AMPA2 receptor and anti-CASPR2 antibodies. Most of the patients were positive for anti-NMDAR antibody (χ^2^ = 72.429, *P* = 0.000).

Regarding the 153 patients with anti-NMDAR antibody positivity, 146 (95.42%) were CSF positive, and 123 (80.39%) were seropositive. The titer for the antibody is shown in Table 1. CSF testing was more sensitive than serum testing (χ^2^ = 16.264, *P* = 0.000), and the antibody titers were higher in the CSF than in the serum (χ^2^ = 16.264, *P* = 0.000). Among the 14 patients with anti-GABA_B_R antibody positivity, 13 (92.86%) were CSF positive, and 13 (92.86%) were seropositive. There was no difference in the positivity rate or titer of anti-GABA_B_R antibody between CSF and serum. Among the 9 LGI1-positive patients, 6 (66.67%) were CSF positive, and 8 (88.89%) were seropositive. The positive rate of serum was higher than that of CSF, but the difference was not significant. Among the 5 CASPR2-positive patients, 1 (20.00%) was CSF positive, and 4 (80%) were seropositive. Although the number of samples was small, the anti-CASPR2 positivity rate of serum was significantly higher than that of CSF.

**Table 1:** Details of antibody titers in the CSF and serum of one antibody-positive patient.

		**Negative**	**Weakly positive**	**Positive**	**Strongly positive**
NMDAR	CSF	7	6	112	28
	Serum	30	17	98	8
GABA_B_R	CSF	1	2	7	4
	Serum	1	4	7	2
LGI1	CSF	3	2	4	0
	Serum	1	1	7	0
CASPR2	CSF	4	0	1	0
	Serum	1	4	0	0

Concurrent positivity for two types of antibodies was found in 8 patients. Further details on double-positive cases are shown in Table 2. Among the patients with LGI1 + CASPR2 positivity, 1 was strongly positive for CASPR2 in the CSF, whereas the antibody titers in the other patients were weakly positive or positive. In addition, only one of the five patients with anti-CASPR2 antibody positivity was CSF positive. All five patients with anti-CASPR2 antibody positivity were also positive for other antibodies, and only one of the five patients was CSF positive for anti-CASPR2 antibody. Presumably, the rate of CSF positivity for anti-CASPR2 antibody is low among AE patients in general.

**Table 2:** Details of antibody titers in the CSF and serum of patients who were positive for two antibody types concurrently.

**Antibodies**	**Cases**	**Antibody titer**
NMDAR + GABA_B_R	Case 1	CSF NMDAR: weakly positive GABA_B_R: positive Serum GABA_B_R: positive
	Case 2	CSF NMDAR: positive GABA_B_R: positive Serum NMDAR: positive GABA_B_R: positive
	Case 3	CSF NMDAR: positive GABA_B_R: positive Serum NMDAR: positive GABA_B_R: positive
LGI1+ CASPR2	Case 1	CSF LGI1: positive CASPR2: strongly positive Serum Negative
	Case 2	CSF Negative Serum LGI1: weakly positive CASPR2: positive
	Case 3	CSF Negative Serum LGI1: weakly positive CASPR2: positive
NMDAR + CASPR2	Case 1	CSF NMDAR: positive Serum NMDAR: weakly positive CASPR2: weakly positive
AMPA2 receptor + CASPR2	Case 1	CSF AMPA2 receptor: positive Serum AMPA2 receptor: weakly positive CASPR2: weakly positive

### Analysis of Factors Correlated With Disease Severity

#### Differences in Disease Severity Between the Sexes

We found that there were differences between males and females with regard to comorbid tumors and prognosis (Table 3). Male patients had tumors in 6 cases (8.22%), and female patients had tumors in 5 cases (4.31%). In the past, it was believed that women were more likely than men to have tumors as a comorbidity with AE, especially given the strong relationship between teratoma and anti-NMDAR encephalitis. However, in our study, male AE patients were more likely than females to have tumors, and 1/2 of males with tumors had GABA_B_R-positive pulmonary tumors. Fifty-six male patients (76.71%) had a good prognosis, and 105 female patients (92.92%) had a good prognosis; thus, more women than men had a good prognosis, and the difference was significant (χ^2^ = 6.766, *P* = 0.009). Among the patients who died, 3 were (4.11%) male and 2 (1.72%) female. Although the mortality rate of males was higher than that of females, there was no significant difference (χ^2^ = 0.990, *P* = 0.376). Moreover, despite being more susceptible than males to AE, females had a better prognosis.

**Table 3:** Details regarding disease severity indicators in males and females.

	**Male**	**Female**
ICU (no.)	25	41
Ventilator use (no.)	9	9
Tumor (no.)	6	5
Surgery (no.)	1	2
Median hospitalization days	21	23
Median hospitalization costs (USD)	4622.27	5005.11
**Prognosis**		
GOS ≥4	56	105
2–3	14	9
1	3	2

#### Differences in Disease Severity Among Different Age Groups

We found that there were differences in antibody distribution, combined tumors, ICU occupancy, and ventilator use between adults and children (Table 4). There were 34 AE cases with non-NMDAR antibody positivity (37.78%) in adults and 2 cases (2.02%) in children. Therefore, non-NMDAR antibody-positive encephalitis was more common among adults than children (χ^2^ = 38.272, *P* = 0.000). There were 11 adult patients and no child patients with cancer; accordingly, adults were more likely than children to have tumors (χ^2^ = 12.848, *P* = 0.000). Additionally, a greater number of adults (56, accounting for 62.22% of all adults) than children (fewer than 10, accounting for 10.10% of all children) were admitted to the ICU. This difference was statistically significant (χ^2^ = 65.253, *P* = 0.000). Among adults, 13 used ventilators, accounting for 14.44% of all adults; among children, 5 used ventilators, accounting for 5.05% of all children. Overall, adult patients used ventilators at a higher rate than children did (χ^2^ = 4.828, *P* = 0.028).

**Table 4:** Details regarding disease severity indicators in different age groups.

	**Adult**	**Children**
ICU (no.)	56	10
Ventilator use (no.)	13	5
Tumor (no.)	11	0
Surgery (no.)	3	0
Median hospitalization days	22	22
Median hospitalization costs (USD)	5005.11	4623.35
**Prognosis**		
GOS ≥4	80	81
2–3	8	15
1	2	3

#### Relationship Between Antibody Titer and Disease Severity

Because the antibody status of the double-positive patients was complex, those 8 out of 189 patients were excluded from this subgroup analysis. Among the 181 patients with single antibody positivity (Table 5), correlation analysis showed that the antibody titer in the CSF was positively correlated with ICU admission (rs = 0.234, *P* = 0.002), with ventilator use (rs = 0.254, *P* = 0.001), and with the presence of tumors (rs = 0.200, *P* = 0.007). There was also a positive correlation between CSF antibody titers and prognosis, but it was not significant (*P* = 0.135). In addition, our analysis found that the serum antibody titer was negatively correlated with ICU admission (rs = −0.329, *P* = 0.000). Conversely, there was no significant correlation between the serum antibody titer and other indicators, such as ventilator use and prognosis.

**Table 5:** Details regarding disease severity indicators in relation to different antibody titers in the CSF and serum.

	**CSF**				**Serum**			
	**Negative**	**Weakly positive**	**Positive**	**Strongly positive**	**Negative**	**Weakly positive**	**Positive**	**Strongly positive**
ICU (no.)	4	5	29	19	21	16	19	5
Ventilator use (no.)	0	1	7	9	6	2	6	2
Tumor (no.)	0	0	5	5	3	0	6	1
Surgery (no.)	0	0	1	2	1	0	1	1
Median hospitalization days	14	19.5	22	30	21	20	23	24.5
Median hospitalization costs (USD)	2439.89	5041.56	4676.74	6391.01	4802.69	4919.42	4700.65	4535.96
**Prognosis**								
GOS ≥4	13	9	107	24	28	22	95	8
2–3	2	1	14	6	5	2	15	1
1	0	0	3	2	2	1	3	1

## Discussion

Since Dalmau et al. (10) proposed the condition of anti-NMDAR encephalitis, an increasing number of AE-related autoantibodies have been detected and used for clinical diagnosis. To date, however, there has been no large-scale epidemiological survey of AE in adults and children, and its epidemiological characteristics remain unclear. In 2016, *The Lancet Neurology* published diagnostic criteria for AE, emphasizing the diagnostic significance of AE-related autoantibodies (5). The epidemiological characteristics of 189 patients with AE and autoimmune antibody positivity in the CSF or serum were retrospectively analyzed in this study.

The most commonly detected synaptic receptor antibodies in Southwestern China are the anti-NMDAR, anti-AMPA1 receptor, anti-AMPA2 receptor, anti-GABA_B_R, anti-LGI1-related, and anti-CASPR2-related antibodies. In the present study, 457 patients with suspected or confirmed AE were examined for serum and CSF antibodies. The positivity rate for antibodies was 41.36%. In a study by Lai et al. (11), 35.78% of patients with AE were positive for antibodies.

The rate of good prognosis was 85.19%, and the mortality rate was 2.65%. This finding is consistent with previous studies, indicating that the overall prognosis of AE is good. One study of 571 patients with anti-NMDAR encephalitis by Kayser et al. (12) reported that 83% of the patients recovered completely or partially. However, in a study by Yeshokumar et al. (13), the mortality rate was 12%; the rate of good prognosis was only 53%, and the rate of poor prognosis was 34%. The differences in the results of that study and of ours may be due to differences in prognosis prediction. The previous study scored prognosis using the modified Rankin scale (mRS), whereas we used the GOS. Moderately disabled patients were classified in our study as having a good prognosis, while the study by Yeshokumar et al. classified such patients as having a poor prognosis. Some scholars have found that among all cases of encephalitis, AE has an especially poor prognosis, with 56% of AE patients dying or having severe disabilities (2, 3). The reason why patient prognosis in previous studies differs so greatly from that in the present study may be that the prognosis of AE associated with tumors is worse than that of non-tumor-related AE. As our study did not involve paraneoplastic AE, the overall prognosis was good.

In terms of the time distribution of AE, there is no previous literature specifying the incidence by season. This study found that autumn (September-November) was the most common season, accounting for 31.22% of all cases, although there was no significant difference among seasons.

With regard to sex distribution, women (61.38%) were significantly more likely than men (38.62%) to have AE. This sex distribution is consistent with previously reported statistics for AE (13). Some possible mechanisms are that estrogen enhances humoral immunity (14) and that mutation of X chromosome-linked genes leads to differential expression of pathogenic genes, leading to the occurrence of autoimmune diseases (15, 16).

In our analysis of age distribution, we observed that the majority of patients were under 45 years old (83.07%). This result is consistent with reported data on other autoimmune diseases in children and young adults. In a study by Titulaer et al. (17), 577 patients had antibody-confirmed anti-NMDAR encephalitis, 95% of whom were under 45 years old, and 37% of whom were children. However, the effect of age on autoimmune diseases is unclear. Some studies suggest that the connection may be related to changes in hormone levels after middle age and that the decrease in estrogen in females weakens the immune response (14).

From 2012 to present, the number of confirmed cases of AE has increased annually, which may be related to increasing awareness of AE among clinicians. In 2007, Dalmau et al. (10) first reported anti-NMDAR encephalitis and found that it was closely related to teratoma. In 2009, Lai et al. (11) reported anti-AMPA receptor encephalitis for the first time, presenting 10 cases associated with type 1 and type 2 glutamate receptors. In 2010, Lancaster et al. (18) first reported and described GABA_B_R encephalitis and found that it may be associated with small cell lung cancer. In the same year, Lai et al. (19) first reported anti-LGI1 antibody-related encephalitis and anti-CASPR2 antibody-related encephalitis associated with voltage-gated potassium channels in *The Lancet Neurology*. Since then, a variety of neuronal autoantibodies have been reported. According to the results of our study, the number of confirmed cases of non-NMDAR encephalitis is also increasing yearly, which may be related to the expansion of the known neuronal autoantibody spectrum and the gradual adoption of antibody detection.

In this study, 189 patients were diagnosed with AE and were antibody positive. The majority were positive for anti-NMDAR antibody (80.95%), followed by anti-GABA_B_R (7.41%), LGI1-related (4.76%), CASPR2-related (2.65%), NMDAR+GABA_B_R (1.59%), LGI1+CASPR2 (1.59%), NMDAR+CASPR2 (0.53%), and AMPA2 receptor+CASPR2 antibodies (0.53%). The distribution of antibodies in this study is fundamentally consistent with that of previous studies. In a study by McCracken (20), 78.82% of patients were positive for antibodies against NMDAR, followed by GABA_B_R (4.71%), LGI1 (4.71%), CASPR2 (2.35%), and others (9.41%). In addition, Guan et al. (21) found 12.9% of 4,106 encephalitis patients to be positive for anti-NMDAR antibody, 12.8% for anti-LGI1 antibody, 5.6% for anti-GABA_B_R antibody, 1.3% for anti-CASPR2 antibody, and 0.6% for anti-AMPA receptor antibody.

The present study found that CSF detection was 15.03% more sensitive than serum detection for patients with anti-NMDAR encephalitis and that the antibody titer in the CSF was higher than that in the serum (*P* = 0.000), which was consistent with previous studies. For example, Gresa-Arribas et al. (9) found that in NMDAR encephalitis, CSF was a more sensitive sample type than blood for detecting anti-NMDAR antibodies. By comparing matched serum and CSF samples, Titulaer et al. (17) also found that the detection sensitivity of CSF was ~15% higher than that of serum for anti-NMDAR encephalitis. However, there was no difference in sensitivity or antibody titer between serum and CSF in other types of antibody-positive AE.

In addition, although the number of CASPR2 antibody-positive patients was small, only 5 cases were CSF positive, and simultaneous detection of CASPR2 and other antibodies in the CSF was found for only one of the five patients. Thus, the positivity rate for CASPR2 in the CSF appears to be low. Bien et al. (22) found that 5 (33.33%) of 15 patients with CASPR2 receptor encephalitis were positive for anti-CASPR2 antibody in the CSF. The sensitivity of the CSF in the previous study was higher than that observed in the present study.

When we analyzed factors related to disease severity, we found that, despite significantly outnumbering male patients, female patients had a better prognosis. In a study by Harutyunyan (23), 16 (59.26%) of 27 AE patients admitted to the ICU were male whereas 11 (40.74%) were female, suggesting that males have more severe disease than females. Additionally, Murphy et al. (24) reported that when BXSB mice developed spontaneous lupus syndrome as an autoimmune disease, males tended to exhibit more severe clinical symptoms than females; the average survival time of male mice was also significantly shorter than that of female mice. Subramanian et al. (25) found that the Toll-like receptor 7 (TRL7) gene, which is related to immunogenesis and development, was heterotopic from the X chromosome to the Y chromosome and was overexpressed, which may explain the autoimmune symptoms in male mice.

The present study found that adult patients with AE are more likely than children to suffer from non-NMDAR encephalitis and to have tumors. Previous studies have shown that anti-AMPA receptor encephalitis may be associated with thymoma, small cell lung cancer, and breast cancer (19, 26, 27); anti-GABA_B_R encephalitis was also associated with small cell lung cancer in another report (18). These reports were consistent with the three cases of GABA_B_R encephalitis with lung tumors found in this study. Anti-LGI1 antibody-related encephalitis and anti-CASPR2 antibody-related encephalitis may also be associated with thymoma (19, 28). In general, adults are more likely to have non-NMDAR encephalitis because they have a higher incidence of tumors than children have (29).

In our analysis of the relationship between antibody titers and disease severity, we found that antibody titers in the CSF were positively correlated with ICU admission, ventilator use, and tumors, which reflect the severity of the disease. Gresa-Arribas et al. (9) have also indicated that antibody titers are higher in severe and teratoma patients. In addition, there was no direct correlation between CSF antibody titers and prognosis in our study, whereas Gresa-Arribas et al. (9) reported that the CSF and serum titers of patients with a poor prognosis were higher than those of patients with a good prognosis. Regardless, Broadley et al. (30) believe that the relationship between CSF titer and prognosis is not exact.

Our study has the following limitations. First, the sample size of the present study was insufficient. Nonetheless, as the number of cases is small for certain low-incidence types of AE, such as anti-CASPR2 antibody-related encephalitis, it is difficult to carry out a statistical analysis. Second, many more autoimmune antibodies are currently tested than the six antibodies mentioned in this paper. However, from 2012 to 2018, the main antibodies detected in Southwestern China were the six described herein. Therefore, we evaluated only these six antibodies. Additional antibodies could be assessed in follow-up studies.

## Conclusion

AE is an inflammatory disorder of the brain that has very complex clinical manifestations and is difficult to diagnose. In recent years, the number of confirmed cases of AE has been increasing annually. Early intervention is very important to improve the prognosis of patients. Neuronal autoantibodies are often a key diagnostic basis in the diagnosis of AE. In our study, 41.36% of patients with suspected AE tested positive for antibodies, and their overall prognosis was good. Women outnumbered men in our sample. There were slightly more children than adults, and children and young adults accounted for the vast majority. Anti-NMDAR encephalitis accounted for the majority of cases; for this type, the sensitivity of antibody detection was higher in the CSF than in the serum, and the antibody titer was also higher in CSF than in serum. It is worth mentioning that the positivity rate for the anti-CASPR2 antibody was higher in serum than in CSF in anti-CASPR2-positive encephalitis cases, with or without concurrent positivity for other antibodies. Analysis of the factors related to the severity of the disease showed that the prognosis of women was better than that of men, that adults were more likely than children to suffer from non-NMDAR encephalitis, and that adults were more likely than children to have tumors. CSF antibody titers were positively correlated with ICU admission, ventilator use, and tumor complications, which may reflect the severity of the disease. However, there was no direct correlation between CSF antibody titers and prognosis. Understanding the epidemiological characteristics of AE can help increase the speed of diagnosis and improve the prognosis of AE patients.

## Author Contributions

All authors listed have made a substantial, direct and intellectual contribution to the work, and approved it for publication.

### Conflict of Interest

The authors declare that the research was conducted in the absence of any commercial or financial relationships that could be construed as a potential conflict of interest.
